# Building a Secure Biomedical Data Sharing Decentralized App (DApp): Tutorial

**DOI:** 10.2196/13601

**Published:** 2019-10-23

**Authors:** Matthew Johnson, Michael Jones, Mark Shervey, Joel T Dudley, Noah Zimmerman

**Affiliations:** 1 Center for Biomedical Blockchain Research Icahn School of Medicine at Mount Sinai Redwood City, CA United States; 2 Institute for Next Generation Healthcare Icahn School of Medicine at Mount Sinai New York City, NY United States

**Keywords:** blockchain, geolocation, tutorial, mobile health, privacy, DApp, iOS, biomedical research, decentralized application, smart contract

## Abstract

Decentralized apps (DApps) are computer programs that run on a distributed computing system, such as a blockchain network. Unlike the client-server architecture that powers most internet apps, DApps that are integrated with a blockchain network can execute app logic that is guaranteed to be transparent, verifiable, and immutable. This new paradigm has a number of unique properties that are attractive to the biomedical and health care communities. However, instructional resources are scarcely available for biomedical software developers to begin building DApps on a blockchain. Such apps require new ways of thinking about how to build, maintain, and deploy software. This tutorial serves as a complete working prototype of a DApp, motivated by a real use case in biomedical research requiring data privacy. We describe the architecture of a DApp, the implementation details of a smart contract, a sample iPhone operating system (iOS) DApp that interacts with the smart contract, and the development tools and libraries necessary to get started. The code necessary to recreate the app is publicly available.

## Introduction

### Background

Decentralized apps (DApps) are computer programs that run on a distributed computing system. These have been popularized recently by distributed ledger technologies underlying projects such as Bitcoin and Ethereum. Unlike the client-server architecture that powers most internet apps, DApps interact with app logic deployed on a blockchain enabling transparent, verifiable, and immutable records of each transaction. When built on blockchain networks, DApps can contain their own suite of associated smart contracts that are used to encode business logic and allow persistent storage of state [1].

Over 150 blockchain projects in the health care industry alone have raised more than US $660 million in private and blockchain-funded markets [2,3]. Despite this massive investment in blockchain technologies over the last 3 years, the DApp ecosystem remains immature. At the time of writing, the most popular DApp had 5628 daily active users [4]. By comparison, Facebook, a popular centralized app, reported 1.5 billion daily active users in Q4 of 2018 [5]. Documentation and tooling for developers to build on new platforms is sparse [6,7]. As a result, the technical hurdles currently required to develop a DApp restrict more widespread experimentation.

Self-contained sample projects [6-10] can help jump-start development, allowing those new to the field to focus on the logic and app instead of the myriad technical decisions required to get started. This tutorial too walks through the development of a complete working DApp prototype, including smart contract and iPhone operating system (iOS) client apps, and is specifically motivated by a real use case that requires data privacy in biomedical research. All of the code necessary to recreate this app has been made publicly available [11,12]. Our hope is that this project will be forked, remixed, and combined to catalyze the ecosystem of biomedical blockchain DApp development.

This tutorial is organized as follows: (1) a discussion around the traditional approach to data privacy in research as well as the rationale for a blockchain approach; (2) the motivation for a geolocation sharing use case for biomedical research; (3) an overview of the software architecture proposed, including a brief description of security properties; (4) a description of the client app; (5) a description of the smart contract; (6) details on the development environment; (7) details on the deployment of the DApp; and (8) a discussion of advantages, limitations, and future directions.

The aim of this paper is to serve as a tutorial for developing a working prototype of a DApp (as shown in the demo video in Multimedia Appendix 1) and to highlight the specific benefits of using a DApp as a method for participants in research to share features of their raw data, while preserving the participant’s privacy by not revealing the raw data itself.

### Traditional Data Protection and Its Shortcomings

Traditionally, data collected in research is managed using a database that is implemented in a client-server network architecture. In this architecture, a database is connected to a backend server, which can then be accessed by researchers (clients). An example of this architecture is an iOS app, functioning as the front end (client), which makes calls to backend code and the database (server) [13]. This database requires some central authority, typically the researcher or research organization, to set permissions and control access resulting in a centralized app. It is the responsibility of the researchers to ensure the privacy and protection of the collected data; this architecture is convenient for researchers in that regard, as they technically have full autonomy over all the data they collect.

Researchers having full access and control over all collected data from participants, however, is not necessarily in the best interest of either party. This approach can place undue burden on researchers who are only interested in nonidentifiable features of the data and would rather not bear the liability of collecting and managing data that is unnecessary to their analysis. On the contrary, participants must blindly trust that the researchers will responsibly manage and protect their raw data, which, by malice or negligence, is not always the case. This becomes increasingly concerning as longitudinal research and high-frequency data collection is becoming more prevalent, exposing participants to greater privacy risks [14].

Although the reduction of data collection and management liability is realizable in any traditional hosted environment, the party hosting the environment will still have access to the raw data. To avoid this, one could employ data minimization and perform all data-processing and feature extraction on the client so that the host never has access to the raw data itself.

For example, researchers could be interested in the points in time that the heart rate of participants went above 60 beats per minute (bpm). Rather than recording, posting, and storing all the heart rate data of participants, the heart rate could be preprocessed on the participant’s mobile device for heart rate data above 60 bpm. However, this would eliminate the ability for researchers or future collaborators who are interested in heart rate data above 70 bpm to use the previously collected data as there is no way to determine the heart rates for the previously collected time points. To collect new heart rate data above 70 bpm would require an update to the client app.

Unlike the traditional, centralized approach of data privacy where the backend code and database are on a centralized server, DApps have backend code running on a decentralized peer-to-peer network, such as a blockchain network [13]. When deployed on a blockchain, the backend code is referred to as a smart contract. A smart contract is what the client uses to interact with a blockchain network. DApps that are built on smart contracts offer unique advantages over centralized client-server alternatives [15] and in the right situations can serve as a trusted intermediary for data management between participants and researchers. As further detailed in Table 1, transparency, autonomy, immutability, and self-sufficiency are some of the core features of blockchain technology and therefore DApps.

As smart contracts are transparent and immutable, it is possible to verify and guarantee the behavior when the smart contract source code is made publicly available by comparing the binary code of the deployed contract against the open-source code. This may, in part, alleviate the participants’ concern that their data could be mishandled or mismanaged. On the contrary, there are no guarantees that traditional database implementations do not secretly collect and log information, or that the data access policies remain private for all time.

Researchers can rely on smart contracts to self-execute for example, to return relevant features of interest from participant data without the researcher having any awareness of the raw data. This allows for the collection of more raw data, while still limiting the exposure of these data to the researcher. In the heart rate example mentioned earlier, all of the heart rate data could be posted to a smart contract, which can be written in such a way that would allow participants to control access to these data. For example, participants could update researcher access from limiting access to heart rate data above 70 bpm to allowing access for heart rate data above 60 bpm. Using only data minimization, researchers would not be able to access previous heart rate data above 60 bpm. Using smart contracts, researchers could achieve data minimization and reduction of liability, while maintaining a degree of flexibility when accessing private data.

**Table 1:** Smart contract properties: benefits and trade-offs.

Property	Description	Benefits	Trade-offs
Transparent	The state of the app is public and inspectable.	App functionality can be audited and validated; Public nature of code incites collaboration.	Requires careful implementation to avoid exposure of sensitive data; Vulnerabilities can more easily be identified and exploited.
Autonomous	Can be designed and deployed such that it does not require any further interaction with the agent that deployed it.	No need for middleman or external arbiter. No external control or manipulation of app behavior.	No customer service: transactions cannot be reversed, and corrections cannot be made.
Immutable	The code defining the contract cannot be modified.	Guarantees that data policy will not change.	Cannot update smart contract with security fixes; requires new contract deployment.
Self-sufficient	Has the ability to coordinate and incentivize resources, in the form of tokens, to execute functions.	A deployed contract stays deployed on blockchain; does not require developer to pay for maintaining a server.	Lack of control over deployed malicious contracts; users of the smart contract pay transaction costs.

It is important to note that there are several alternative approaches that strive to provide guarantees of data privacy preservation. Many of these approaches rely on trusted third parties, cryptographic, secure hardware, or blockchain-based techniques and are actively being researched and are under development. A full examination of these approaches is out of scope for this tutorial, but a forthcoming article that accompanies this study provides a detailed comparison of techniques based on ability to preserve data privacy and on the practicality of implementation [16]. One of the primary findings is that blockchain-based techniques, particularly when combined with other techniques such as secure hardware, can provide high levels of data privacy, verifiability, and practicality of implementation.

### Use Case: Sharing Location Data for Biomedical Research

This tutorial presents a mobile iOS DApp that allows a research study participant (*participant*) to share useful features of their location data derived from global positioning system (GPS) coordinates, or geocoordinates, with a research team (*third party*), without revealing their raw GPS coordinates. Location data have proven diversely useful in biomedical research where it has improved disease management and treatment delivery, been used to monitor behavioral and environmental risk factors, and even has informed public health policy in substance abuse [17-20].

Although geolocation data hold significant promise for a variety of health care apps, location data are also one of the most sensitive pieces of personal information [21]. It is in the interest of both the researchers and the research participants that the collection of these data has been restricted to the stated goal of the research project, and this study aims to show that blockchain technology can be deployed in such a way to attain these ends.

### Decentralized App Architecture and Use

**Figure 1:**
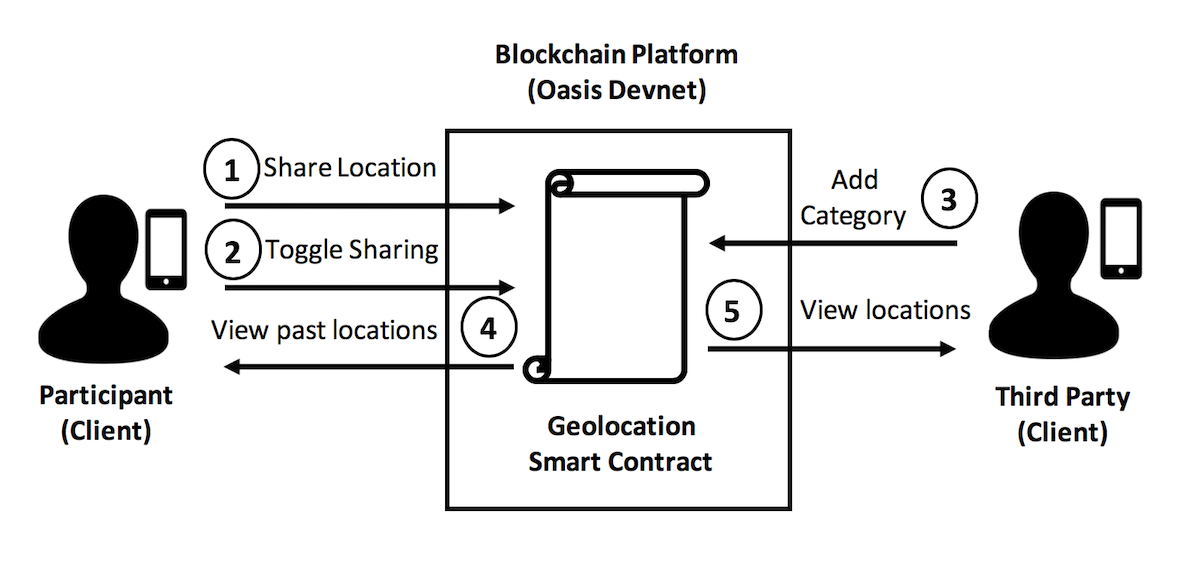
Decentralized application architecture and workflow—Smart contracts consist of self-executing code run on a blockchain protocol. Data flow directly between the smart contract and the clients: (1) Participant submits timestamped geolocation data; (2) Participant grants/revokes permission to share that data, to the smart contract; (3) A third party assigns geolocations of interest a matching category (ie, hospital, gym, pharmacy, or none) and deploy that mapping to the smart contract; (4) Participant can view the timestamp of each of their previously written geolocations and the category of that geolocation, if there exists a mapping between that geolocation and a category that was previously written to the smart contract by a third party; and (5) A third party can view timestamped data that the participant has chosen to share.

The DApp consists of a *client app* for the participant and the third-party researcher and a *smart contract* (Figure 1). A smart contract is a collection of code and data that encapsulate the business logic of the DApp [1]. Smart contracts are written in a high-level programming language, compiled into bytecode, and deployed to a unique address on a blockchain. The development and execution of smart contracts are supported by a variety of blockchain platforms such as Ethereum, EOS, Tron, POA, and Oasis [4,22]. Each blockchain implementation offers different features and trade-offs based on their purpose and protocol; therefore, it is important to consider the features when considering a blockchain platform solution. For additional blockchain related terms and definitions mentioned throughout this study, see Multimedia Appendix 2.

As shown in Table 2, we compare features of a traditional database against Ethereum, a popular public open-source blockchain platform, and against Oasis Devnet, a privacy-preserving blockchain solution.

Data privacy is critical when dealing with biomedical data. However, most public blockchains lack confidentiality and privacy of state variables and data. Smart contracts deployed on Ethereum, for example, allow for the state and data stored within them to be read by anyone. Therefore, public blockchains that lack data privacy and confidentiality would not be a suitable standalone solution for storing and handling sensitive biomedical data [23,24].

The Oasis Devnet addresses this critical gap in blockchain’s lack of confidentiality by combining blockchains with trusted execution environments (TEEs) [25]. The Oasis Devnet is based on Ekiden, a system anchored in a formal security model expressed as a cryptographic ideal functionality [25,26]. The underlying blockchain in Oasis is encrypted, which prevents the dedicated storage of the contract data and state to be read, unlike public blockchains. Within Ekiden, anyone with a TEE-enabled platform can participate as a compute node to execute contracts within the contract TEE. To create or retrieve the keys required to run the contract, the contract TEE must reach out to the key management committee, a quorum of compute nodes that manage the keys needed to run the contract. This system prevents a malicious node from forking the blockchain and acting as a compute node, as they would not have the necessary keys from the key management committee to run the contract.

Additional technical details involving the security and privacy-preserving features underlying the Oasis platform can be found in the study by Cheng et al [25].

Therefore, we selected the Oasis Devnet to deploy this DApp because it (1) prioritizes privacy preservation, (2) has a functional and supported developer network, and (3) is compatible with the Ethereum toolkit.

**Table 2:** Features of traditional databases compared with Ethereum and the Oasis Devnet.

Features	Traditional database	Ethereum (public)	Oasis Devnet
Data read access control	Yes	No, data is public	Yes, dependent on smart contract logic
Anonymity	Yes, if host is honest	Pseudo-anonymous	Pseudo-anonymous
Cost	Fixed	Variable	Free on developer network, will be variable in production mainnet
Data privacy	Yes, if host is honest	No, data is public	Yes, dependent on smart contract logic
Data mutability	Mutable, but can be immutable via role permissions	Immutable	Devnet gets reset, but the production mainnet will be immutable
Code can be updated	Yes	Yes	Not yet. Intercontract calls are planned
Publicly verifiable	No, the public cannot verify stored procedures	Yes, the public can verify smart contract code^a^	Yes, the public can verify smart contract code^a^
Widely accessible	Yes	Yes	Yes

^a^For both Ethereum and Oasis Devnet, the smart contract source code must be made public to verify that the contract is doing what is claimed.

The Oasis Devnet supports several runtimes, allowing developers to choose between popular languages. Here we use Solidity, an object-oriented programming language for writing and implementing smart contracts, because of its existing development frameworks and interoperability with Ethereum, the second largest blockchain network [27]. It should be noted that the Oasis Devnet was used, as the mainnet was not yet available at the time of this writing. In contrast to an open testnet, the Oasis Devnet is hosted by Oasis Labs and was created specifically for developers to make an easy-to-use, developer friendly environment to develop and test on Oasis [28].

Using a blockchain that supports private states, such as Oasis, allows the handling of raw data to shift from that of a central party to a blockchain platform that provides a combination of transparency and privacy via verifiable smart contracts and a system by which no party can view or access the raw data.

### Client App

To simplify the tutorial codebase, the iOS app can toggle between a participant mode and a third-party mode; in practice the two views would be implemented as separate apps that only display the functionality relevant to that user type. Multiple participant users and third-party researcher users could use the client app at once, depending on the desired enrollment and collaboration goals of the research study design.

#### Participant Mode

**Figure 2:**
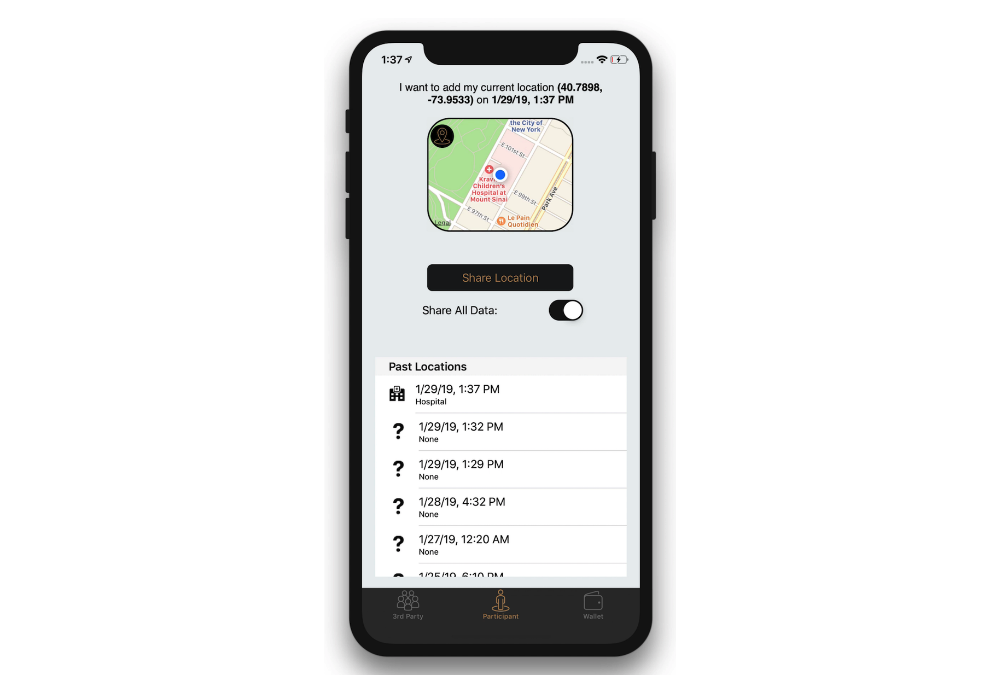
Simulator running the iPhone operating system app displaying the participant mode.

The participant tab presents a participant user with the common functionalities and user interface that a participant in a research study would expect (Figure 2). These include posting the user’s current location, toggling data sharing, and viewing the user’s previously posted data.

This view also presents the user with the device’s geocoordinates and local time to display the human-readable data that are posted to the smart contract, should the user decide to share their location. The geocoordinates are represented as the latitude and longitude of the device with a precision of 4 decimal degrees, approximately 11.132 meters at the equator. This precision was chosen to provide sufficient granularity between establishments, without requiring several adjacent coordinates to represent a single establishment. When posting to the smart contract, these geocoordinates are multiplied by a factor of 10^4^ to represent the decimal degrees as signed integers. If the user has not previously posted a location, a new participant identifier (ID) for the user is created, and the associated sharing status is enabled and set to true.

The participant can toggle whether or not to continue sharing their current and previous data anytime. If sharing is enabled then all third parties are able to view the number of previously submitted locations by the participant, along with the timestamped category. If sharing is disabled, third parties can only see that participant’s ID and that they have chosen not to share data at this time. A design decision was made to make the default sharing status opt-out, for 3 reasons: (1) The primary participant user action is to “share location,” which itself informs the participant that their data will be shared; (2) Participant data privacy is provided regardless of the sharing status, but is available because we believe participants own their data and should have the ability to revoke access; and (3) To maximize benefit to third parties who want to access to participant location feature data.

A third party can benefit from seeing the ID of participants that have revoked data access to better understand how many participants exist and how many of them continue to share data over time. Showing all participant IDs was included primarily as a demonstration to easily show how access can be granted and revoked; however, the smart contract could be easily written in such a way that no longer allows third parties visibility into the participant IDs who have revoked access. This may be an important security consideration in scenarios where there are few participants. As additional participant IDs are revealed to third parties, regardless of sharing status, this could provide some level of data to malicious third parties that may attempt to extrapolate a participant’s wallet address from the participant ID by examining the history of transactions with the smart contract. Fortunately, the method that is called by a transaction is concealed, so there is no direct means of differentiating between a participant and a third party based on examining the transaction history.

The participant is also presented with a table containing previously posted locations ordered by the time posted. Each location displays its corresponding category and timestamp. These previously posted locations will always be visible to the participant that posted them, even if sharing is disabled at that time. This allows a user to view the data that will be shared if they choose to do so.

#### Third-Party Mode

**Figure 3:**
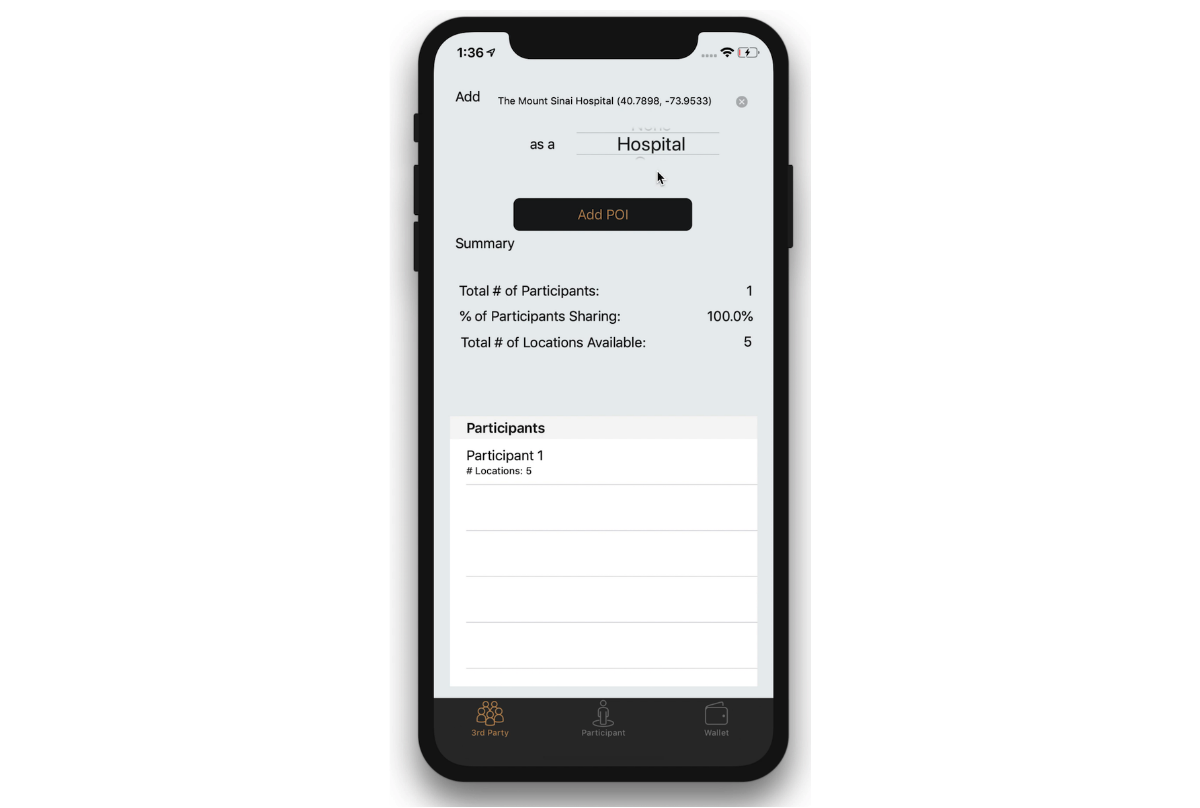
Simulator running the iPhone operating system app displaying the third-party mode.

The third-party view presents views and functionality for a third party, such as a research coordinator (Figure 3). These include categorizing a geolocation and viewing participant feature data.

Within this view, a third-party user is able to search for map-based addresses and places of interest. The places of interest are centered around the location of the device, through Apple’s MapKit Framework, where the geoencoding service is performed by Apple. It should be noted that the geoencoding service is done solely as a convenience to the third party and is not done for the participant nor identifies the participant’s location. Once the user has selected a location and selected its associated category, they can post to the smart contract.

The view also presents a summary of participant data, such as the number of participants who have posted at least one location, the percentage of participants who have enabled sharing, as well as the total number of locations that are available and being shared. For specific information on each participant, the third party is shown a table view of all participants and whether the participant has enabled the sharing of their data. If a participant has enabled the sharing of their data, third parties are able to view how many locations a participant has posted and can view the timestamp and location category of each entry.

### Smart Contract

The smart contract manages participant enrollment in the research study and allows participants to share their geocoordinates with the study. The smart contract also determines whether the geocoordinates correspond to a predefined location type and whether or not to allow third parties to view the data participants commit to the smart contact. Importantly, raw geolocation data are not stored within the smart contract, nor are the geolocation data directly linked to the participant.

### Decentralized App Development

#### Tools and Libraries

Many tools and libraries exist for the development of DApps. The tools and libraries used to develop this DApp are briefly introduced and summarized and will be expanded upon further throughout the discussion of development (Table 3). Certain resources in this tutorial are platform-dependent, such as Oasis Contract Kit, whereas others are generally useful for DApp development such as ConsenSys AG’s MetaMask and Truffle.

**Table 3:** Decentralized app–related resources used in this tutorial.

Tools and Libraries	Descriptions
MetaMask	Browser extension that serves as a Web3 wallet that can create and manage identities. It also injects the web3.js library into the browser to allow read and write requests to be made on blockchain networks, such as Ethereum or other networks by specifying a remote procedure call URL.
Oasis Contract Kit	Docker environment with a preconfigured set of tools for developing contracts on Oasis.
Oasis Devnet	Privacy-focused blockchain platform for developers to build and test confidential smart contracts; the platform used in this tutorial.
Remix	Web browser–based integrated development environment that allows developers to write, deploy, and run smart contracts written in Solidity.
Solidity	An object-oriented programming language for writing and implementing smart contracts on various blockchain platforms.
Truffle Framework	A development environment testing framework and asset pipeline for blockchains using the Ethereum Virtual Machine; included in Contract Kit.
Web3swift	Open-source iOS library written in Swift. It provides web3.js functionality in Swift, native ABI parsing, and smart contract interactions.

#### Client App

The client is a native iOS app, written in Swift 4.2 using XCode 10.1. The iOS app interacts with the smart contract deployed on the Oasis Devnet via the open-source web3swift library written by Matter, Inc. This library allows for the interaction with a remote node of the Oasis Devnet via JavaScript object notation (JSON) remote procedure call (RPC) as well as smart contract interaction [29]. The web3swift library also provides local keystore management, which assists the app user in creating and importing a wallet as well as the creation and management of public/private keys. This eliminates an additional burden and barrier to entry for users unfamiliar with wallets and key management. When implementing the web3swift library, the web3 instance was bound to the RPC URL provided in the Oasis documentation [22]. The contract instance within the iOS app was initialized from the app binary interface, or ABI, string in JSON format and was obtained from the Ethereum Foundation’s Remix integrated development environment (IDE) or whichever IDE was used to develop the smart contract. The ABI is a data encoding and decoding scheme and is the standard way to interact with smart contracts in Ethereum for interfacing with smart contracts.

It should be noted that as the client app is a proof-of-concept, there are many improvements which can and should be made in production. Currently, none of the data read from the smart contract are persistently stored on the device, which hinders performance. Similarly, none of the raw geolocation data are persistently stored on the device. If any data were to be persistently stored, it should be encrypted. Currently, the only data that are persistently stored on device includes the public wallet address, whether that address has been registered, and the encrypted private key of said address with a user provided password via AES-128-CBC. In practice, access to data should require authentication whenever the app is left in an inactive state for a given amount of time.

#### Smart Contract

The smart contract is developed in Remix, a Web browser–based IDE that allows developers to write, deploy, and run smart contracts written in Solidity [30]. Remix allows for contract deployment in various types of environments, including: a JavaScript virtual machine (VM) in which transactions are executed in a sandbox blockchain in the browser, an Injected Provider in which Remix will connect to an injected Web3 provider such as MetaMask, and finally a Web3 provider in which Remix will connect to a remote blockchain node [31]. Initially, the smart contract was developed in the JavaScript VM environment in Remix for its simplicity.

**Figure 4:**
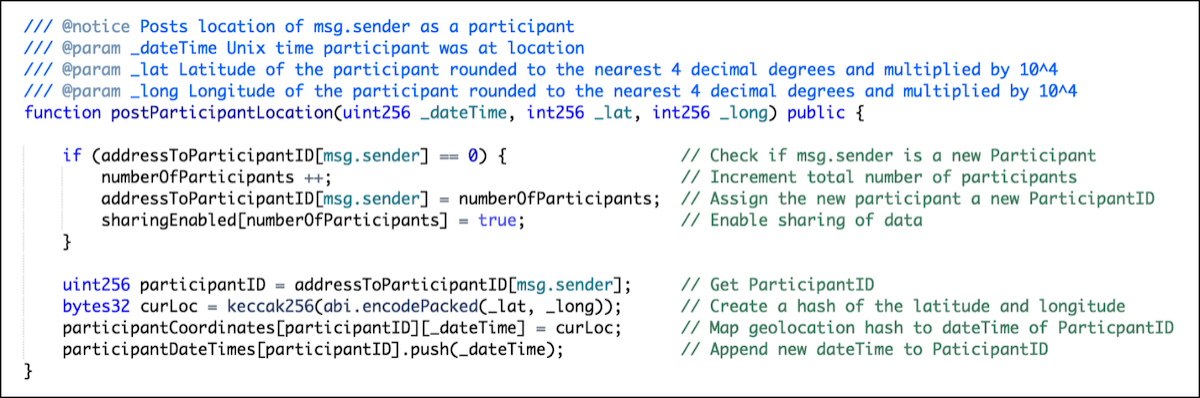
Solidity method written to post the location of the participant. This method (postParticipantLocation), when called by the client, (1) checks to see if the sender is a new participant by checking to see if there exists a mapping for the sender’s address to an existing participant ID (addressToParticipant). (2) If one does not exist, the total number of participants is incremented (numberOfParticipants). (3) The senders address is then mapped to a participant ID that is equal to the count of participants.

Figure 4 illustrates how raw geolocation data are not stored within the smart contract and also not directly linked to the participant. This design was selected because Oasis Devnet offers Ethereum backward compatibility with support for existing Ethereum wallets. This allows for existing Ethereum wallets to be imported into the app or for newly created wallets within the app to also be used on Ethereum. Although convenient, this could result in the exposure of identifying information on the Ethereum network being effortlessly correlated to the same address on the Oasis Devnet, should the same address be used on both networks. Therefore, a participant’s wallet address is not revealed to third parties, and instead, third parties have access to the participant ID.

Considering that geolocation data should not be directly linked to the participant, the participant ID is used to identify a participant. Once the sender address has been assigned and mapped to a participant ID, the keccak256 function is used to compute the Ethereum-SHA-3 (Keccak-256) hash of the latitude and longitude input parameters (*_lat, _long*) as a convenient encoding. This hash is stored as a private variable, not accessible outside of the smart contract, and later used to uniquely identify this particular geolocation within the smart contract. The hashed geolocation is mapped to the timestamp input parameter (*_dateTime*), which is subsequently mapped to the respective participant ID. This hashed geolocation is also used to compare with other hashed geolocations, to determine if that particular geolocation matches a category type posted by a third party. The timestamp input parameter (_*dateTime*) is then appended to a mapped array of the participant ID (*participantDateTimes*). This allows third parties to assign a category to a particular geolocation. It is important to note that the privacy of these input variables, hashed geolocations, and mappings within the smart contract are made possible by the Oasis Devnet as it supports private data that can only be accessed by the smart contract themselves. Descriptions of additional variables and functions within the smart contract can be found in Multimedia Appendices 3 and 4.

A similar method exists for both storing a mapping of the particular category type to the Ethereum-SHA-3 (Keccak-256) hash of the geolocations and storing a mapping of an array of the hashed locations to the category type.

It is worth noting that these category types were deliberately predefined as *enum* types on contract creation to prevent third parties from creating and storing new and custom categories that could be used to identify particular locations (eg, by posting a custom category that labels the accompanying geolocation with a postal address or geocoordinate pair.) This provides a way for third parties to add new locations that match the predefined category types into the future, whereas at the same time holding them responsible for the transaction cost of adding these new locations.

### Decentralized App Deployment

#### Wallet and Funding

Before the deployment of the smart contract onto the Oasis Devnet, a hierarchical deterministic (HD) wallet was created and funded from the Oasis faucet. MetaMask was installed and set up to be used as this wallet [32]. When creating the wallet, the mnemonic phrase, or seed words, used to generate the HD wallet was safely stored and made easily accessible, as it was later needed for deploying the contract. Once the wallet and account were created, the network within MetaMask was changed to the custom RPC URL, chainID, symbol, and nickname provided by Oasis Labs to connect MetaMask to the Oasis Devnet [28].

Funds were acquired via the Oasis Devnet Faucet by following the onscreen instructions [33]. Once the funds were received, the wallet within MetaMask updated to show the amount funded in DEV. DEV, the symbol for Oasis Devnet tokens, were used to pay the transaction fees needed to deploy the contract [22]. DEV have no value and work only on the Oasis Devnet, as they will not transfer to the Oasis mainnet [22]. It was important that the first account created by MetaMask was the one that was funded, otherwise deployment of the contract to the Oasis Devnet would have failed. If a different account was funded by mistake, DEV could have been transferred from the funded account to the first account.

#### Contract Deployment

The contract was deployed using Oasis Contract Kit. The Oasis Contract Kit is a Docker environment with a preconfigured set of tools to provide developers with an environment that provides tools for developing, testing, and deploying confidential smart contracts. Following the steps provided by Oasis, the geolocation smart contract was deployed as both a confidential and nonconfidential smart contract on the Oasis Devnet (procedure described in Multimedia Appendix 5) [12,34].

#### iPhone Operating System App and Deployed Smart Contract

To interact with the nonconfidential smart contract, the iOS app was set, by default, to initialize a web3 instance using the RPC URL provided by Oasis, as well as initialize an instance of the deployed smart contract using the ABI and contract address. Unlike Ethereum and other standard Web3 platforms, transactions to confidential smart contracts on the Oasis platform are encrypted end-to-end, where only the caller and the smart contract can decrypt transaction contents [35]. Communication with the Oasis Devnet occurs via hosted nodes by Oasis, and the client iOS app uses HTTPS, an encrypted communications protocol using Transport Layer Security, to protect data in transit. Oasis also provides a web3c.js client library that wraps underlying RPC calls with encryption and decryption, which allows one to securely communicate with smart contracts. Due to a lack of web3swift support for web3c, a web3 extension for confidential transactions, the iOS app was unable to interact with the deployed confidential smart contract at the time of this writing. Although the nonconfidential implementation is sufficient for the purposes of a working demonstration, it should be stressed that the nonconfidential implementation does not provide the participant any privacy of their posted location data.

#### Creating/Importing a Wallet

If there is no stored wallet on the first launch of the app, the user is presented with an option to create a new wallet or to import an existing wallet. When creating a wallet, the user is prompted to enter a user-generated password, which is later used to access the wallet private key and perform transactions that cost DEV, such as posting participant location data. Once set, the user-generated password cannot be recovered so it is recommended that it be written down or stored in a safe place. In the case of importing a wallet, the user would use their previously recorded password and would also have to provide their private key either manually keying it in or, for convenience, using the device’s camera to scan a QR code encoded with the private key. Once a wallet is in place, the user will be presented with 3 tabs: *third party*, *participant*, and *wallet* [36].

#### Funding the Wallet

**Figure 5:**
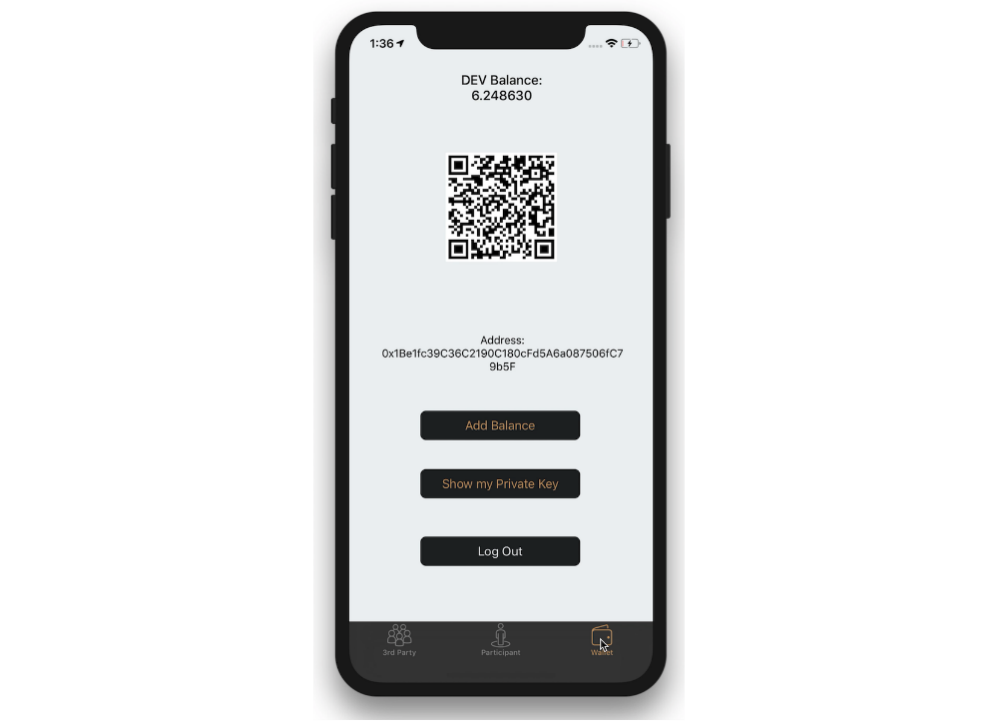
Simulator running the iPhone operating system app, displaying the user’s wallet information.

The wallet view presents the user with views and functionalities associated with managing a wallet on a blockchain and is required for both user types (Figure 5). These include allowing the user to view their wallet address and providing a link to fund the wallet. Regardless of whether the user is a third party or participant, the user would first have to add funds to their wallet, which is done within this tab. To make any changes to the state of the smart contract, such as a participant posting their geolocation or a third party assigning a new geolocation a location type, the user is still required to have funds to pay the transaction fees.

To add funds, a user would first navigate to the wallet tab to obtain their wallet address. For convenience, a button has been added that will copy the wallet address to the clipboard and open the URL for the Oasis Devnet Faucet in the default Web browser [33]. Once the user fills out their information to request funds for the Oasis Devnet, they will receive a confirmation email. Upon email confirmation, the user can return to view the updated balance of DEV for their wallet address. Within this tab, the user can also find a QR code with their embedded wallet address. As a security measure, any function resulting in a state change to the contract requires password reauthentication to gain access and view their wallet’s private key. If the device is lost or the app deleted from the device, the only way to recover the wallet and access to the account is to import the private key.

## Discussion

To assess DApps and blockchain as a potential solution to the pitfalls of traditional data privacy preservation in research, a geolocation sharing use case was proposed. An iOS DApp and smart contract were developed as a proof-of-concept to illustrate the advantages and disadvantages that accompany this approach.

Although data privacy in research has typically been managed by a trusted third party, there are key areas in which this centralized approach falls short. Participants must trust third parties/researchers to properly use their data but also trust that their data are protected and secure from others who may misuse the data. As blockchains present an alternative method for data management and app logic that does not rely on a trusted third party, there is an opportunity where DApps could be explored as an alternative method for preserving data privacy.

### Advantages

#### Smart Contract Properties

Smart contracts allow for transparency as the code can be made public and easily verified against the deployed smart contract (Table 1). As a result, users can trust that a validated smart contract does what it claims to do, such as not revealing raw location data in our case, proving it is able to act as a trusted intermediary for data management between participants and third parties. This public and open nature invites collaboration, allowing others to test the smart contract as well as share code of their own to improve the DApp community. Smart contracts are immutable; thus, participants can be confident that once a smart contract used by the DApp is verified and proven to be secure, the resulting management of data will also remain unchanged for the lifetime of the contract.

#### Costs

With traditional client-server architecture, there is a fixed cost in maintaining a backend database. Even if the database is no longer in use, or hardly ever accessed, there is still typically a cost for keeping it functioning and accessible. With a DApp, however, the only costs associated with hosting are the transaction cost of deploying the smart contract and the individual user costs incurred when modifying states within the contract.

An example of a state change in the example DApp is when a participant posts their location data to the smart contract. This is incurred because the smart contract is storing new data, and the state of the contract has changed. However, viewing data that exists in the smart contract can be done at no cost to a third party; for example, viewing how many participants are in a study. Depending on how the smart contract is written, viewing data could also come at a cost. This can be particularly useful in aligning and incentivizing different users toward a mutually beneficial goal. In the example DApp, the smart contract could be written in such a way that third parties pay a small fee, in DEV, to the participant, to view the category of each location that the participant has visited. This may incentivize more participants to share more data with third parties.

#### Access Control

This DApp illustrates how a third party that is interested in a particular feature, such as a location category, could determine the feature without accessing any raw data. Limiting the scope of data shared with a third-party research team helps protect the participant’s data from being misused outside the context of the study [14].

If, at any time and for any reason, the participant decides to stop sharing feature data with the third party, they are able to revoke third-party access to their data. As the source code governing the smart contract can be made public and can be verified to prove that the contract executes as claimed, participants can be assured that access to third parties is in fact revoked. Traditional databases, however, could continue to provide access to third parties unbeknown to a participant as access control cannot be publicly verified. Empowering the participant with control over their data may encourage participation and sharing of data by participants by explicitly addressing data privacy concerns.

#### Participant Identity

One advantage to using this DApp is the ability for the smart contract to create and maintain a private mapping between the participant ID and the participant’s wallet address. This is advantageous as only the smart contract has access to this identifying and sensitive data, thus further safeguarding participants’ identities. This also obviates the need for a separate party to manage the pseudonymization of the participants’ identities.

#### Data Exposure

Traditional approaches may use external parties and services to perform feature extraction in an attempt to limit exposure to the raw location data. In this example, a third party may use a service, such as Google Places, to accomplish categorizing past locations. If such a service were used, that service could collect, store, and share a participant’s raw geocoordinates and potentially identify the participant unbeknown to the third party or participant. Even if the raw location data is anonymized before using these external services, this increases privacy risks as it has been shown that deanonymization based on location data is possible [37,38]. This DApp provides an alternative to external services by potentially sourcing multiple interested third parties who are able to contribute to the mapping of locations to particular categories. Utilizing a DApp to perform feature extraction on the raw data provides one less potential entity in possession of participants’ raw data.

### Disadvantages and Limitations

#### Smart Contract Properties

Although there are several advantages to this DApp framework, there are also several limitations. Making smart contracts public and verifiable inherently allows for the discovery and exploitation of vulnerabilities. Should vulnerabilities be discovered, the smart contract cannot be updated in the same way a backend server would; instead it would require the deployment of a new smart contract. Smart contracts cannot be changed once deployed, so mistakes made by either a participant or third party cannot be reversed or corrected, except in the case where it was written into the smart contract. In blockchains that offer cross-contract calls, such as Ethereum, an upgradable smart contract design could be implemented. This design would offer the ability to deploy new smart contract versions by redirecting the contract address, thus allowing an upgradable contract. However, smart contracts within Oasis are unable to interact with one another at the time of this writing, not making it possible to implement in the context of the Oasis Devnet. In the future, however, Oasis plans to add support for cross-contract calls [39]. Additional design considerations during smart contract development can be found in Multimedia Appendix 6.

#### Transaction Costs

To make any state changes to the smart contract such as a participant posting their location or a third party posting a new location, the DApp user must pay a transaction fee. To pay this fee, the DApp user must have a minimum amount of funds in their wallet, which requires an additional task of obtaining funds. In the case of this DApp, the network is the Oasis Devnet, the fee is paid for in DEV, and funds can be requested free from the Oasis Devnet Faucet [33]. It is important to note that at the time of this writing, obtaining funds for use within the Oasis Devnet requires identifying information from the user; however, it is assumed that this is only during the early stages of development of the Oasis platform.

#### Onboarding and Usability

Most users are unfamiliar with activities required for DApp interaction, such as wallet creation, private key storage, and obtaining tokens. As DApps and smart contracts are built on blockchain technology, this requires user actions that are not required by traditional client-server architecture. As the technology matures, we anticipate these activities will become more streamlined, similar to how these functions and interactions have matured on the World Wide Web. Blockchain DApp transactions can appear quite slow in comparison with traditional client-server architecture, but this is an issue that is actively being worked on by the blockchain community.

#### Malicious Users and Decentralized App Design

DApp users are identified by their wallet’s unique public key. Public blockchains, on which most DApps are built, are typically not permissioned, and so anyone can create an infinite number of wallets and unique public keys. As users are able to remain pseudonymous and hide their true identities by proxy of their unique public key, this makes it difficult to ban or blacklist malicious users from accessing the DApp. Similarly, without being able to verify a user’s true identity, a malicious user is able to falsely act as another type of user. For example, a malicious participant could pose as a third party or vice versa.

This DApp was designed to allow third parties to continue to add new locations that match the predefined category types into the future. However, these new locations may conflict with previous locations, especially in very dense areas. As the latitude and longitude are rounded to the nearest 4th degree of precision, this results in an accuracy of approximately 11.132 meters with an error of half that distance. Very dense population areas, such as cities, may have multiple categories of location for that particular area. For example, a gym may exist within less than 11.132 meters of a hospital resulting in more than just one possible category per location. To address this, the DApp was designed to allow future posted categories to overwrite previous ones. However, a malicious user acting as a third party can post an incorrect category for a given location and would be able to overwrite a previously posted category. This would allow the malicious user to iterate through a given location area, post a particular category, and then identify the participants’ previous location categories that have changed, which implies that the particular location is the same as that location just posted by the malicious third party. However, because of the transaction costs associated with posting new categories, this iterative process could become prohibitively expensive and thus, may disincentivize this activity. There are also several ongoing blockchain projects working towards the verification of location data in an attempt to combat malicious users [40,41].

### Future Improvements

#### Informed Consent

User onboarding and informed consent are tasks that would require careful design tailored to the particular study and data that are being collected and are out of scope for this tutorial. An informed consent would explain to users that only features of their data were being collected (eg, location category from geocoordinate data) and would take into consideration the legal aspects related to the collection and ownership of data. Although the consent could be managed outside of DApp, a better user experience would be to include a digital informed consent screen within the app, which could also illustrate how the data are used, which parties are involved, and the ability to revoke data sharing.

#### Expanded Data Features

This DApp provides a basic example of feature extraction for the category of the location from the raw GPS data. One could easily envision more advanced feature extraction for this use case using geolocation data. Additional metadata such as the total distance traveled in a day, variance in the number of locations visited, or the travel radius of participants could be computed by the smart contract, if the smart contract were redesigned to securely store the raw geocoordinates of the locations.

#### Additional Data Sharing Use Cases

As the source code of the smart contract is publicly available, the smart contract could easily be adapted and shared to fit a variety of needs beyond geolocation data. Other data commonly stored on iOS devices, such as heart rate and steps walked within Apple’s HealthKit, could be posted to the smart contract and could make features of the data viewable to interested third parties. Moreover, various data streams could be combined and used within the same smart contract to output additional features, all while safeguarding the sensitive raw data used to create them.

This software has been made publicly available on GitHub at HD2i/GeolocationSmartContract and HD2i/Geolocation-iOS. It was the intention of the authors that improvements, via forks or pull requests, would be made to improve this tutorial. Our goal with this tutorial is to inspire new and unforeseen improvements that would help advance the community as a whole [11,12].

Through this simple use case we have intended to highlight the potential of privacy preservation using smart contracts on blockchain networks such as Oasis. It also illustrates how using a native mobile DApp, privacy-preserving smart contracts could be used to ensure the confidentiality of sensitive health data and at the same time provide researchers with the feature-rich data embedded within.

## Appendix

Multimedia Appendix 1 Demo video.

Multimedia Appendix 2 Terms and definitions.

Multimedia Appendix 3 Smart contract state variables.

Multimedia Appendix 4 Smart contract functions.

Multimedia Appendix 5 Steps to deploy geolocation smart contract.

Multimedia Appendix 6 Design considerations.

## Abbreviations

bpm: beats per minute
DApp: decentralized app
GPS: global positioning system
HD: hierarchical deterministic
ID: identifier
IDE: integrated development environment
iOS: iPhone operating system
JSON: JavaScript object notation
RPC: remote procedure call
TEE: trusted execution environment
VM: virtual machine
