# Supplementary material for: Building a Secure Biomedical Data Sharing Decentralized App (DApp): Tutorial
Source: J Med Internet Res. 2019 Oct 23;21(10):e13601. doi: 10.2196/13601 (PMC6835476; doi:10.2196/13601)
Supplement: Multimedia Appendix 2 [file jmir_v21i10e13601_app2.pdf]

Term	Definition
Application Binary Interface (ABI)	A data encoding scheme used in Ethereum to work with smart contracts that determines certain details, such as how functions are called and the type of data that they return.
Client-Server Architecture	Typically used in traditional databases where a user, also known as a client, can modify data which is stored on a centralized server. In this architecture, a designated authority is in control of and provisions access to the database.
Docker	An open-source tool designed to easily create, deploy, and run applications by using containers. Containers package an application with everything it needs to run, such as libraries and other dependencies, and ship it all out as one package.
Faucet	A source to obtain free cryptocurrency or tokens, typically used in developer networks, where the cryptocurrency or tokens hold no monetary value.
Fork	When developers copy source code and independently develop it into a separate piece of software
Hash	A hash function, takes input objects such as strings or integers and outputs a fixed size number or string where this output is typically referred to as a hash. The hash, as it relates in this text, is generated in such a way that it makes it incredibly difficult to determine the input that was used to generate it.
Smart contract	Smart contracts consist of self-executing software code run on top of a blockchain protocol
Transaction Fee	A fee that is charged to users who either perform transactions or change the state of a smart contract.
Wallet	Stores public and private keys, which allow users to perform transactions or interact with smart contracts.
