# Supplementary material for: Building a Secure Biomedical Data Sharing Decentralized App (DApp): Tutorial
Source: J Med Internet Res. 2019 Oct 23;21(10):e13601. doi: 10.2196/13601 (PMC6835476; doi:10.2196/13601)
Supplement: Multimedia Appendix 3 [file jmir_v21i10e13601_app3.pdf]

Variable Name	Description
creator	The address of the creator of the contract
Category	The predefined categories: None, Hospital, Gym, and Pharmacy
numberOfParticipants	A public variable representing the total number of Participants who have posted at least one location
addressToParticipantID	A private mapping of the Participants wallet address to a Participant ID
sharingEnabled	A public mapping of Participant IDs to whether they have enabled sharing with Third Parties
participantCoordinates	Mapping of Participant IDs to a mapping of the date/time of the hash of posted latitudes and longitudes
participantDateTimes	Mapping of Participant IDs to an array of all of the date/times of their posted locations
categoryLocation	Mapping of the category type to an array of all the hashed geocoordinates of locations posted by Third Parties of that category type
locationToCategory	Mapping of the hashed geocoordinates of locations posted by Third Parties to their respective category type
