# Supplementary material for: Building a Secure Biomedical Data Sharing Decentralized App (DApp): Tutorial
Source: J Med Internet Res. 2019 Oct 23;21(10):e13601. doi: 10.2196/13601 (PMC6835476; doi:10.2196/13601)
Supplement: Multimedia Appendix 4 [file jmir_v21i10e13601_app4.pdf]

**Function Name**	**Description**
postParticipantLocation	Posts the timestamp and the hash of a Participant's formatted latitude and longitude. Creates a new Participant ID for the Participant address, if one doesn't exist. Assigns the sharing preference of the new Participant to be true.
postParticipantSharingPreference	Toggles the sharing of the Participant's location data
getParticipantID	Returns the sender their Participant ID
getParticipantSharingStatus	Returns a flag to the sender indicating their current sharing status, if one exists
getParticipantNumberOfLocations	Returns the sender with the total number of locations they have posted
getParticipantDateTimeOfLocations	Returns the Participant with the timestamp, as Unix time, of a particular posted location, given an index
getParticipantCategory	Provides the Participant with the category of a particular posted location, given the posted location's timestamp
postPOI	Assigns a category to a particular set of geocoordinates, given the category and geocoordinates.
getSharingEnabled	Returns the sharing status of a particular Participant
getNumberOfLocations	Returns the number of locations a particular Participant has visited, if that Participant has enabled sharing
getDateTimeOfLocations	Returns the timestamp of a particular posted location for a particular Participant ID
getCategory	Returns the category of a particular posted location given a Participant ID and Unix time of the posted location.
