# Supplementary material for: Building a Secure Biomedical Data Sharing Decentralized App (DApp): Tutorial
Source: J Med Internet Res. 2019 Oct 23;21(10):e13601. doi: 10.2196/13601 (PMC6835476; doi:10.2196/13601)
Supplement: Multimedia Appendix 5 [file jmir_v21i10e13601_app5.pdf]

- Download Contract Kit by running:
  - ‘docker pull oasislabs/contract-kit’
- Launch Contract Kit:
  - ‘docker run -ti –rm -v “$PWD:/project oasislabs/contract-kit bash’
- Within the Contract Kit Environment:
  - Create a new project directory
    - mkdir geolocation && cd geolocation
  - Configure an empty Truffle project
    - ‘truffle unbox oasislabs/oasis-box’
  - Create a new file of type .sol
    - For a non-confidential contract, name it:
      - “Geolocation.sol”
    - For a confidential contract, name it:
      - “confidential-Geolocation.sol”; or
      - “confidential_Geolocation.sol”
  - Copy the contents from the developed contract in Remix and save the new file
  - Compile the contract
    - ‘truffle compile’
  - Open truffle-config.js and add the seed words used to create your MetaMask wallet for the constant variable named *MNEMONIC*.
  - Create a file named ‘migrations/2_geolocation_migration.js’ with the following contents:
    - ```
      const Geolocation = artifacts.require("GeoLocation");
      module.exports = function(deployer) {
        deployer.deploy(Geolocation);
      }
      ```
  - Run the migration to deploy the contract to Oasis Devnet:
    - ‘truffle migrate -- network oasis’
      ```
