# Supplementary material for: Building a Secure Biomedical Data Sharing Decentralized App (DApp): Tutorial
Source: J Med Internet Res. 2019 Oct 23;21(10):e13601. doi: 10.2196/13601 (PMC6835476; doi:10.2196/13601)
Supplement: Multimedia Appendix 6 [file jmir_v21i10e13601_app6.pdf]

Wallet funding and key management

Most users are unfamiliar with activities associated with DApp interaction and so a best attempt was made to alleviate the onboarding pains of new DApp users by handling wallet creation and management within the iOS client. In this tutorial, we leverage the web3swift library to provide the user with the ability to easily create a new wallet, which also handles the management of the associated public/private keys. This eliminates an additional burden and barrier to entry for those unfamiliar with DApp and key management. Similarly, in order to help the user fund their wallet with the tokens necessary to use the DApp, a link to the Oasis Devnet Faucet provided.

Transaction Fees

Another design consideration that should be examined are transaction fees. Making any change to the state of the smart contract requires a transaction fee. This may affect participant engagement, as it requires that the participant to pay a transaction fee for each location they post and every time they want to change sharing preference of their previously posted data. One possible way to alleviate this burden would be to incorporate an incentive structure into the smart contract that would pay the user back for their location contribution; however, this incentive structure results in a financial incentive for dishonest posts by malicious participants. Another possible approach, is to restructure the smart contract and iOS app to allow for the batch-posting of locations so that it would cost slightly less per transaction. Similarly, the hashing of the latitudes and longitudes could be done on the client side and posted to the smart contract to eliminate an additional operation within the smart contract, thus reducing transaction costs. Most of the design considerations of DApps revolve around transaction fees and would depend on the DApp's end users such as participants and third parties.

Access Control

Another limitation is the design pattern that was used for access control in the smart contract, which in this context is defined as selectively releasing secret information to authorized parties under specific conditions. A caller-based access control was used in the smart contract, which at the time of this writing is an experimental feature that is in progress. As explained by Oasis, this is an experimental feature as the sender of a message, or call, can be spoofed, resulting in the release of secret information to someone who is not authorized to view it. This is problematic in the context of this smart contract when participants wish to disable sharing of their previously posted locations' timestamps and categories. A malicious user could potentially spoof the sender of a message, or call, and appear to be a participant who's sharing is currently disabled in order to view that participant's previously posted location's timestamps and categories. However, this can be easily remedied by

switching the smart contract to use an event-based access control pattern, as events can only be decrypted by the user who caused them to be generated.

Malicious users and Permissions

Another design decision was predefining the location categories within the smart contract so that they could not be changed once the contract was deployed. This was done to prevent malicious third parties from adding categories that were uniquely identifiable. However, there could be situations where it would be desirable to allow third parties to add new categories. One way to address this concern is to permission or white-list certain third parties to allow them to add new categories.

In the example of the tutorial DApp, a single malicious user can also generate multiple wallets resulting in multiple participant IDs while in reality only being a single participant. Similarly, without being able to verify a user's true identity a malicious user is able to falsely act as another user. For example, a malicious participant could pose as a third party, or vice-versa. This malicious user can act as a third party, post incorrect categories for given location, and would be able to overwrite previously posted categories. This would allow the malicious user to iterate through a given location area, post a particular category, and then identify which participants' previous location categories have changed implying that the particular location is the same as that location just posted by the malicious third party.

This could potentially be solved by not allowing previously posted categories to be overwritten, at the expense of reducing the flexibility of changing or updating categories of locations in the future. Similarly, a malicious user can act as a participant and submit false information, or more specifically in this case, spoof their current location along with false timestamps. Although such malicious users would be required to pay the transaction fee as a means of deterring such malicious acts, payment of the transaction fee may not suffice particularly in the current Oasis Devnet where DEV holds no fiat value. To address the malicious user posing as third parties and participants, one could incorporate a permissioned system into the contract where a trusted entity such as the contract creator could be given the ability to white-list only trusted addresses to act as DApp users as a means controlling who the users are and whether what they are posting is truthful. However, this would require participants and third parties to trust the additional entity managing the white-listing and tracking of third parties. In regards to the malicious user spoofing their location, verifying data integrity is beyond the scope of smart contract design, but it should still be noted.
